# Case of Fungous Tumor Occupying the Left Maxillary Sinus, Successfully Treated by the Extraction of the First and Second Superior Molares of the Affected Side

**Published:** 1846-06

**Authors:** Chapin A. Harris


### ARTICLE VII.

*Case of Fungous Tumor occupying the Left Maxillary Sinus, Successfully Treated by the Extraction of the First and Second Superior Molares of the Affected Side.* By CHAPIN A. HARRIS, M. D., D. D. S.

MISS L——, of Baltimore, twenty-two years of age, of a bilious temperament, called to consult me in relation to the condition of her teeth, on the 10th of February last. On examination, the crowns of the first and second superior molares of the left side were found badly decayed, and which, from the destruction of the greater portion of their sockets, were much loosened. The gums on either side of them were very much swollen, spongy, and had a livid appearance; from between the edges of which, whenever the teeth were touched, a thin, fetid matter, occasionally streaked with blood and pus, was discharged. She complained of a sensation of fulness and occasionally of slight pain in her left cheek. The affected molares had been troublesome and sensitive to the touch for nearly three years, resulting, as she supposed, from a severe cold, as she suffered about that time, for near two weeks, the most violent pain in them. She had, subsequently, several times, been urged by her friends to have these teeth removed, but the fear of pain had prevented her from submitting to the operation.

Fearing that the diseased condition of the sockets of the affected molares had extended to the antrum maxillare, and confident that the parts immediately involved could not be restored to health so long as they remained in the mouth, I advised her to have them removed. After much persuasion, she consented to submit to the operation.

The gums being separated from the teeth, I at once grasped the first molaris with a pair of forceps, and proceeded to remove it. It readily yielded to a very slight force, but the moment this was applied, a gush of blood issued from the left nostril, and the complete removal of the tooth being prevented by a fungous excrescence which had originated from the extremity of its roots, and passed up into the antrum, the true nature of the affection at once suggested itself to my mind. The tooth, after being partially removed, was liberated by cutting the excrescence.

The hemorrhage for a few minutes was profuse, but after it had partially subsided, the socket was examined, when an opening was discovered through the floor of the antrum, large enough to admit the end of the little finger,—the fungous peduncle, after its separation from the roots of the tooth, having contracted, had passed up into this cavity. This was now partially explored by means of a small probe, and found to be nearly filled with a soft, spongy tumor, which bled profusely from the slightest injury. Finding a portion of the floor of the antrum, back of the tooth which had just been extracted, in a necrosed condition, and partially exfoliated, I extracted the second molaris, which also had a fungous excrescence upon the extremity of its roots, that passed up through an opening from its socket into this cavity, and then removed the dead bone. This occupied the space between the two teeth.

An opening was now formed through the floor of the antrum, of about an inch in length, and more than a quarter of an inch in width, which enabled me to explore the interior of the cavity more thoroughly than I had previously been able to do. The tumor, which at first had completely filled it, had, from the hemorrhage which had been caused by the extraction of the two teeth, become so reduced in size, that I was enabled to pass a small curved probe between it and the walls of the sinus, and by this means to satisfy myself that it had no connection with any part of this cavity. There was no danger, therefore, to be apprehended of a reproduction of the excrescence after its removal, which was easily effected, by piece-meal, with a small, sharp-pointed hook, and a narrow bladed knife.

The opening through the alveolar border into the antrum soon closed, and the parts, in a short time, were restored to a healthy condition.

*Remarks.*—It is well known to dental practitioners, that large fungous excrescences or tubercles sometimes form upon the extremity of the root of a tooth, and that when it occurs on the root of an upper molaris, if the floor of the antrum be very thin, an opening is formed through it, and instead of remaining in the alveolus, it passes up into this cavity, where it occasionally attains a very large size, and this is precisely what had taken place in the case just described. But in this instance, spongy excrescences from two teeth had passed up into the antrum, united, grew until they had filled the entire cavity, and caused a necrosis of a portion of its floor.

What would have been the result, had the teeth been permitted to remain, is not difficult to conjecture. The pressure of the excrescence, as it augmented in size, would have caused a necrosis of the entire floor, if not of the walls of the antrum, which would ultimately have become detached and displaced, carrying it and the diseased teeth with them. But, in the mean time, other parts might have become involved in a worse and more unmanageable form of disease.

